# Efficacy and safety of duloxetine in chronic musculoskeletal pain: a systematic review and meta-analysis

**DOI:** 10.1186/s12891-023-06488-6

**Published:** 2023-05-18

**Authors:** Xuezhi Ma, Shijie Zhou, Wenyuan Sun, Jie Sun, Guangguang Li, Lining Wang, Yang Guo

**Affiliations:** 1 Department of Orthopedics, Affiliated Hospital of Nanjing University of Traditional Chinese Medicine, Nanjing, China; 2 Laboratory of New Techniques of Restoration & Reconstruction of Orthopedics and Traumatology, Nanjing University of Chinese Medicine, Nanjing, China; 3 Yixing Hospital of Traditional Chinese Medicine, Wuxi, Wuxi China; 4 School of Chinese Medicine, School of Integrated Chinese and Western Medicine, Nanjing University of Chinese Medicine, Nanjing, China; 5 Chinese Medicine Centre (International Collaboration between Western Sydney University and Beijing University of Chinese Medicine), Western Sydney University, Sydney, Australia

**Keywords:** Duloxetine, Chronic musculoskeletal pain, Pain, Meta-analysis

## Abstract

**Background:**

Chronic musculoskeletal pain (CMP) is a complex condition that is mainly treated with analgesic drugs. However, antidepressant intervention is also an important factor in the treatment of CMP. Duloxetine is an effective treatment option for patients with CMP as its antidepressant effect. The purpose of this article is to evaluate the efficacy and safety of duloxetine in treating CMP.

**Databases and data treatment:**

We searched PubMed, Web of Science, Embase, Cochrane Library from inception to May, 2022. Randomized controlled trials (RCTs) evaluating the efficacy and safety of duloxetine versus placebo in patients with CMP were included. We identified 13 articles and studied a population of 4201 participants in 4 countries.

**Results:**

This meta-analysis showed that the duloxetine has statistically significant compared with the placebo control, benefits on 24-hour average pain, living quality, physical function, and global impressions and there was no difference in the incidence of serious adverse event. In general, duloxetine can improve mood and pain level at the same time.

**Conclusions:**

This review shows a significant contribution of duloxetine to CMP symptom relief. This meta-analysis improved that duloxetine can significantly reduce the pain level of patients, improve depressive symptoms and global impression, and has no obvious serious adverse reactions. However, additional studies are required to confirm the relationship between psychological diseases and chronic pain and explore their internal links.

**Supplementary Information:**

The online version contains supplementary material available at 10.1186/s12891-023-06488-6.

## Background

Chronic musculoskeletal pain (CMP) is a persistent pain that affects muscles, joints, bones and other parts of the human body. CMP encompasses a wide range of motor system diseases, including osteoarthritis, neck pain, low back pain and fibromyalgia syndrome. About 20-33% of the global population suffers from CMP. With the increasing of population aging and social pressure, the incidence of CMP has increased significantly [1]. In addition to pain and dysfunction, CMP can also lead to depression and psychological disorders in patients. About 43.3% of patients with CMP had depression or bipolar disorder [2]. According to a meta-analysis [3], individuals who experience chronic pain report higher depression than those healthy controls. Long-term chronic pain and psychological disorders greatly reduce patients’ quality of life while imposing a significant psychological and economic burden on patients and their families.

Currently, treatment of CMP relies on analgesic drugs such as non-steroidal anti-inflammatory drugs (NSAIDs), antipyretics, opioids, and muscle relaxants. However, NSAIDs will increase the risk of peptic ulcer, gastrointestinal bleeding, cardiovascular adverse events and have poor efficacy [4–6]. Antipyretics such as acetaminophen also have a risk of acute liver failure [7, 8]. Opioids have strong analgesic effects but are prone to serious adverse reactions such as dependence, abuse and addiction [9]. For patients with CMP and psychological disorders, analgesic drugs have limited therapeutic effects. Therefore, finding a relatively safe and effective drug is an urgent problem in the process of making CMP medical decisions.

Duloxetine, a potent and selective serotonin and norepinephrine reuptake inhibitor (SNRI), is the only antidepressant drug approved by Food and Drug Administration (FDA) to treat CMP [10]. Duloxetine can resist depression, anxiety and adverse psychological emotion, and it can also inhibit the release of excitatory neurotransmitters, blunt the nociceptive pathway, and play a comprehensive effect on CMP. Duloxetine has a good safety profile and a low dose for long-term use in patients with chronic pain of different races in a retrospective analysis [11]. There have been meta-analyses on the efficacy and safety of duloxetine for knee osteoarthritis or chronic low back pain [12, 13], but few studies were included. This study conducted a meta-analysis on the efficacy and safety of duloxetine in the treatment of patients with CMP by expanding the sample size and types of diseases. We try to provide relevant basis for the necessity of antidepressant treatment in the treatment of CMP by studying the effect of duloxetine on the mood and psychological state of patients with CMP.

## Methods

### Literature search

We searched PubMed, Cochrane Library, Medline, Web of Science from inception to May, 2022 by using a combination of abstract and key words, such as “duloxetine”, “chronic musculoskeletal pain”, “osteoarthritis”, “fibromyalgia”. We limited the literature what was published but did not place any limits on language or publication date.

### Inclusion criteria and exclusion criteria

The title and abstract were obtained by two independent researchers, and we used EndNote to manage the retrieved literature. After reading the title, abstract and full text, the literature was screened and checked. If there was any disagreement, the third researcher would decide. The literature we included had to meet the following criteria: (1) study type: RCTs of efficacy and safety of duloxetine in the treatment of CMP, such as knee osteoarthritis (KOA) or fibromyalgia; (2) study group: Patients diagnosed as KOA or fibromyalgia and the course of disease was more than 3 months; (3) interventions: Duloxetine was used in the test group, placebo was used in the control group, and NSAIDs were allowed to be used together; (4) the outcome indicators included at least one of the following: Brief Pain Inventory-Severity(BPI-S)、Brief Pain Inventory-Interference(BPI-I)、Western Ontario and McMaster Universities Osteoarthritis Index(WOMAC)、36-Item Short-Form Health Status Survey(SF-36)、Clinical Global Impressions of Severity(CGI-S)、Patients’ Global Impression of Improvement(PGI-I)、Serious Adverse Events(SAEs), and data were extracted at the end of follow-up for each outcome.

### Data extraction

Two researchers (Xuezhi Ma, Shijie Zhou) screened and extracted the data independently, reviewing the title, abstract, and full text of each article, and consulted a third researcher (Yang Guo) when disputations arose. The extracted data mainly includes the following contents: (1) basic information of each study including first author, country, publication year, study design, etc.; (2) age of patients, sample size, interventions, period of treatment; (3) the data of case and control groups; (4) potential sources of biases.

### Quality assessment

We assessed the risk of bias for each study using the items in Cochrane Collaboration’s tool [14] for assessing quality in randomized trials, which included the following items: (1) selection bias included random sequence generation and allocation concealment; (2) blinding of participants, personnel, and outcome assessment; (3) incomplete outcome data; (4) selective reporting; (5) other potential bias. We evaluated all the above biases and divided them into “low, unclear and high bias risks”. On this basis, we used the Grading of Recommendations Assessment, Development and Evaluation (GRADE) tool to further evaluate the level of the included literature, which is divided into “high, medium, low or very low” quality.

### Statistical analysis

In this study, we tried to contact the authors to obtain the original data when the data were missing or incomplete. For data that were not available, the investigators used the evidence-based transformation formula to obtain means and other corresponding data [15]. Pooled mean difference (MD) with 95% confident interval (CI) was calculated for continuous data while relative risk (RR) with 95% CI for dichotomous data. Heterogeneity was assessed using the I^2^ statistic. If I^2^ < 50%, the heterogeneity of articles was considered to be small, and the fixed effects model was used. Otherwise, the random-effects model was used [16]. In addition, sensitivity analysis would also be performed in the case of heterogeneity by eliminating one study at a time, so as to check for the resolution of heterogeneity [17]. Besides, the publication bias was assessed using the visual funnel plot and Egger’s test, with a P < 0.05 indicating significant publication bias [18]. Forest plots were used to display the results from individual studies and pooled estimates, and P < 0.05 were regarded as statistically significant. Data analysis was performed using RevMan 5.3.

## Results

### Study selection

**Fig. 1:**
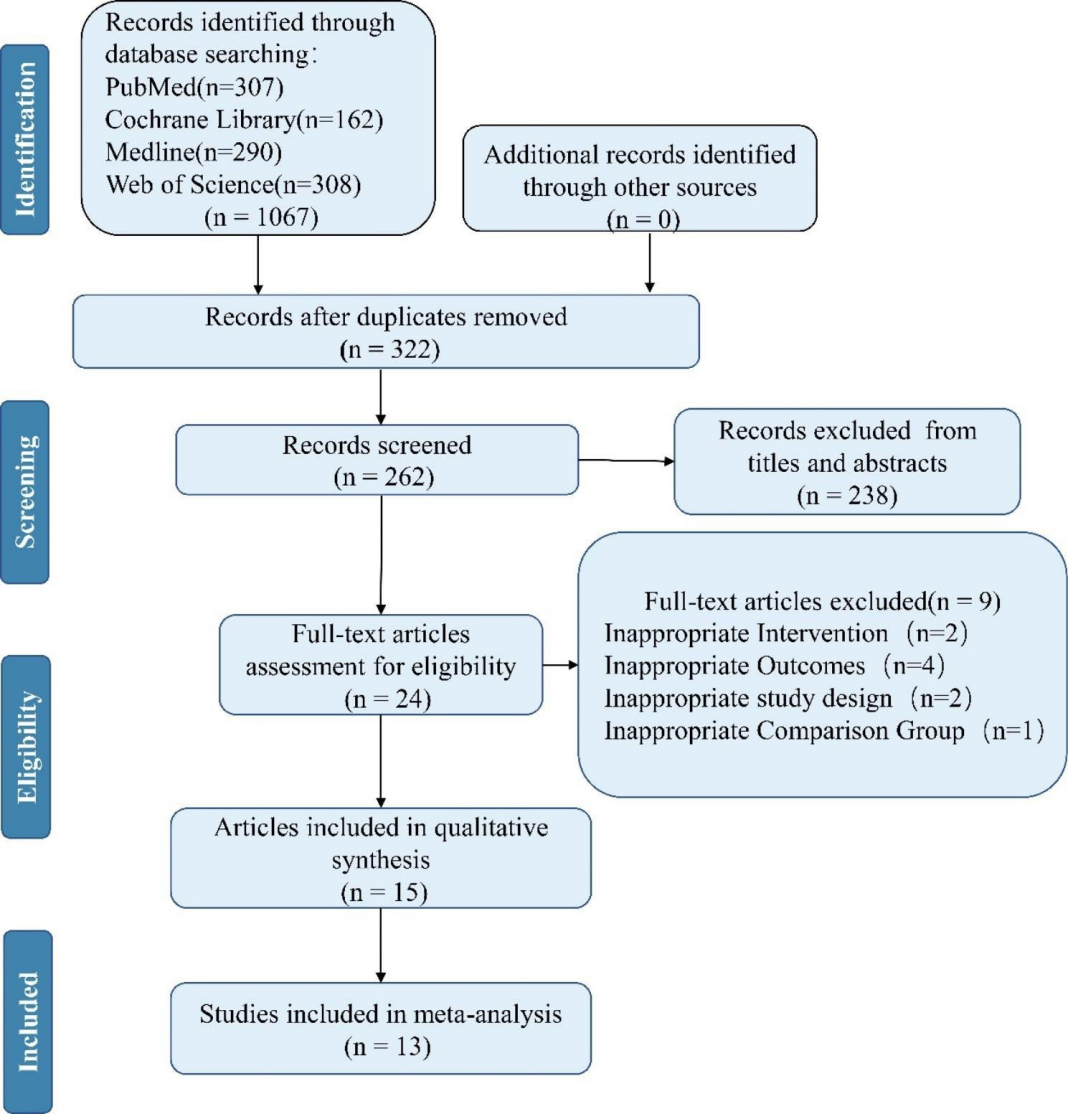
PRISMA Flow chart of study selection

As is briefly illustrated in Fig. 1, 1067 articles were obtained according to the literature retrieval strategy. After deleting the duplicate content and screening the title and abstract, 1043 articles were excluded. 11 articles were excluded due to inappropriate intervention or other reasons. Finally, 13 studies [19–31] from 4 countries met our eligibility.

### Study characteristics

The baseline characteristics and treatment regimens of the 13 eligible studies with a total sample size of 4201 patients are displayed in Table 1. All included studies were compared with placebo, while 6 studies [19, 22–26] were patients with KOA and 7 studies [20, 21, 27–31] were patients with fibromyalgia.

**Table 1:** Baseline characteristics and treatment regimen of included studies

Study	Time	Location	Population	Trial Group			Control Group			Study duration (wk)	Main outcome
				n	Age(y)	Treatment regimen	n	Age(y)	Treatment regimen		
Himanshu	2019	USA	fibromyalgia	91	15.74 + 1.4	Duloxetine 30-60 mg/d	93	15.33 + 1.4	placebo	13	①②⑤⑦
Uchio	2018	Japan	KOA	177	65.5 ± 8.0	Duloxetine 60 mg/d	176	66.4 ± 8.4	placebo	14	①②③④⑤⑥⑦
Wang	2017	China	KOA/Hip	205	61.2 ± 8.2	Duloxetine 60 mg/d	202	59.8 ± 8.4	placebo	13	①②③⑤⑥⑦
Murakami	2015	Japan	fibromyalgia	191	47.8 ± 12.0	Duloxetine 60 mg/d	195	49.5 ± 11.7	placebo	14	①②④⑤⑥⑦
Abou-Raya	2012	Egypt	KOA	144	68.9 ± 6.2	Duloxetine 60 mg/d	144	68.5 ± 5.8	placebo	16	③
Arnold	2012	USA	fibromyalgia	155	50.9 ± 11.9	Duloxetine 30 mg/d	153	50.7 ± 12.5	placebo	12	①④⑤⑥⑦
Frakes	2011	USA	KOA	264	61.6 ± 9.2	Duloxetine 60-120 mg/d + NSAIDs	260	60.3 ± 9.2	placebo + NSAIDs	10	①②③⑤⑥⑦
Chappell	2011	USA	KOA	128	63.2 ± 8.8	Duloxetine 60-120 mg/d	128	61.9 ± 9.2	placebo	13	①②③④⑤⑥⑦
Arnold	2010	USA	fibromyalgia	263	50.7 ± 11.3	Duloxetine 60-120 mg/d	267	49.6 + 10.8	placebo	24	①④⑤⑦
Chappell	2009	USA	KOA	111	62.1 ± 9.6	Duloxetine 60-120 mg/d	120	62.5 ± 9.3	placebo	13	①②③④⑤⑥⑦
Russell	2008	USA	fibromyalgia	147	51.5 ± 10.8	Duloxetine 120 mg/d	144	50.3 ± 10.9	placebo	24	①④⑤⑥
Arnold	2005	USA	fibromyalgia	116	49.6 ± 10.9	Duloxetine 120 mg/d	120	49.6 ± 10.9	placebo	12	①②④⑤⑥⑦
Arnold	2004	USA	fibromyalgia	104	49.9 ± 12.3	Duloxetine 120 mg/d	103	48.3 ± 11.3	placebo	12	①④⑤⑥

Main outcome: ①BPI-S, Brief Pain Inventory-Severity;②BPI-I, Brief Pain Inventory-Interference;③WOMAC, Western Ontario and McMaster Universities Osteoarthritis Index;④SF-36,36-Item Short-Form Health Status Survey;⑤CGI-S, Clinical Global Impressions of Severity;⑥PGI-I, Patient’s Global Impression of Improvement;⑦SAEs, serious adverse events

Abbreviations: KOA, Knee osteoarthritis; NSAIDs, non-steroidal anti-inflammatory drugs

### Quality assessment

**Fig. 2:**
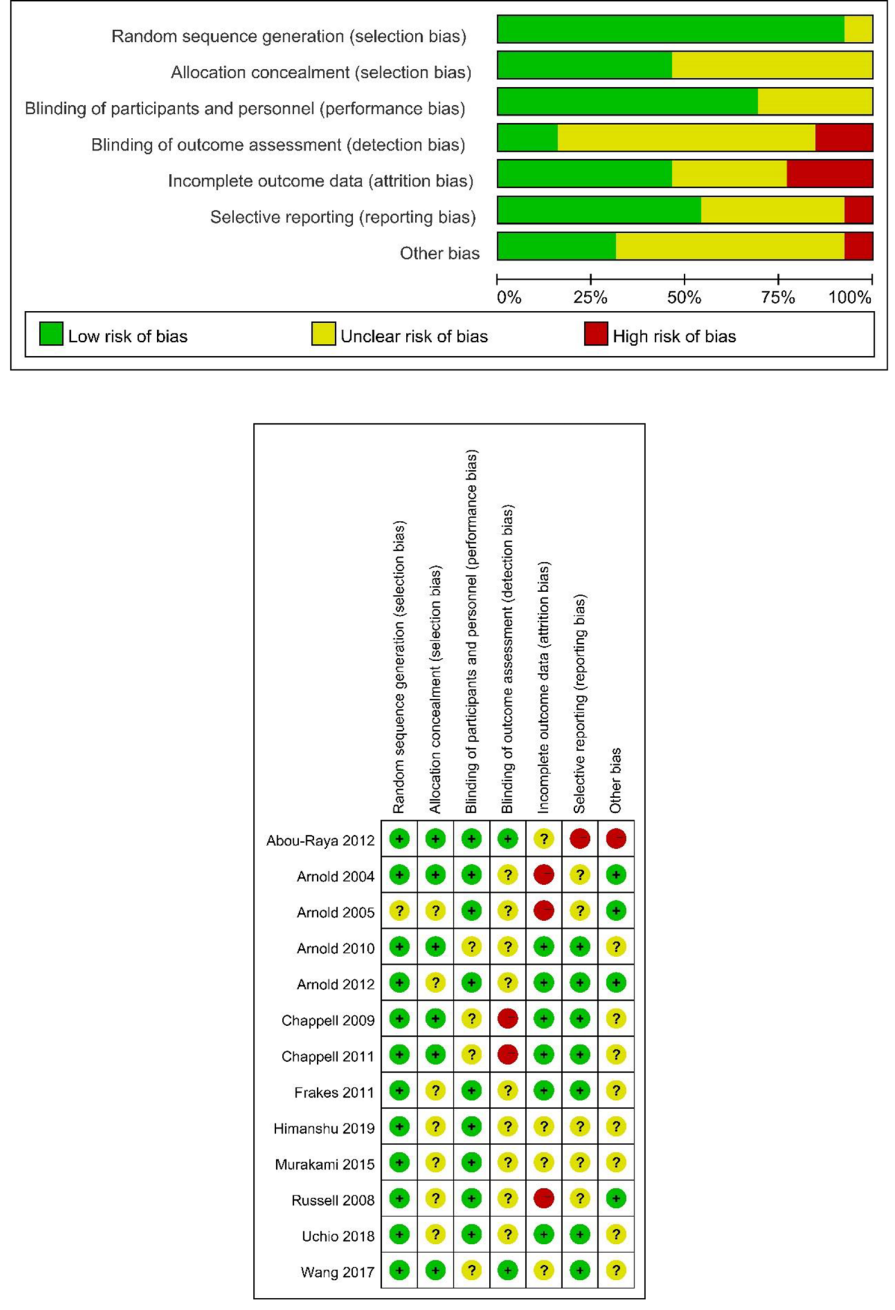
Risk of bias summary and assessment summary of randomized controlled trials (green = low risk; red = high risk; yellow = unknown)

The quality assessment of the trials was performed using the Cochrane Collaboration’s risk-of-bias tool. Two studies [25, 26] explicitly described the stochastic methods, and the rest of the studies [19–24, 27–31] just mentioned “random”. None of the studies described detailed allocation concealment processes. Blinding of participants and personnel occurred in all 13 studies. In general, all studies we included had a low risk of bias. The detailed results are presented in Fig. 2.

### Meta-analysis results

In the 13 eligible RCTs, 12 trials [19–22, 24–31] measured pain level using BPI-S and 8 trials [19–22, 24–26, 30] assessed living quality by BPI-I. Six trials [19, 22–26] evaluated the physical function using WOMAC physical function and stiffness subscale. Some trials compared the patient’s global impression measured by CGI-S [19–22, 24–31] and PGI-I [19, 20, 22, 24–28, 30, 31]. Ten trials [19–22, 24–26, 28–30] of all reported the numbers of serious adverse reactions (SAEs).

### Effect of duloxetine on pain relief

**Fig. 3:**
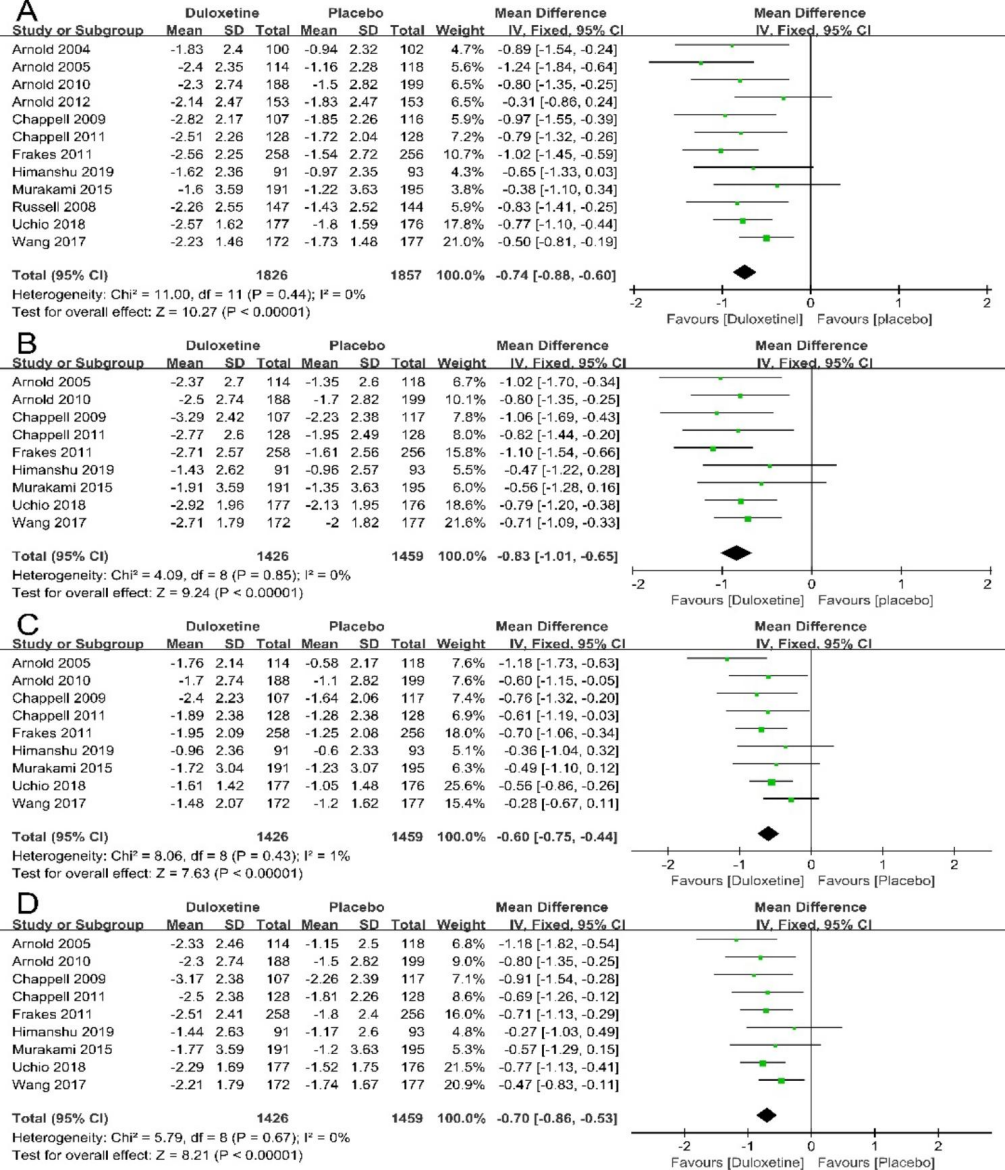
Forest plot: Effects of duloxetine on pain measured by BPI − S: (A) average pain; (B) worst pain; (C) least pain; (D) pain right now. The black horizontal lines represent the 95% confidence intervals, while the squares represent the point estimate. The black diamond represents the overall point estimate and 95% confidence intervals

Twelve trials contributed to the meta-analysis of pain relief based on the BPI-S. Compared with the placebo control groups, the meta-analysis results indicated that patients in the duloxetine groups had significant reductions in the average pain within 24 h (12 articles; 3683 patients; MD= -0.74; 95% CI, -0.88 to -0.60; P<0.00001) (Fig. 3A), worst pain (9 articles; 2885 patients; MD= -0.83; 95% CI, -1.01 to -0.65; P<0.00001)(Fig. 3B), least pain (9 articles; 2885 patients; MD= -0.60; 95% CI, -0.75 to -0.44; P<0.00001) (Fig. 3C) and pain right now (9 articles; 2885 patients; MD= -0.70; 95% CI, -0.86 to -0.53; P<0.00001) (Fig. 3D). These studies suggest that duloxetine significantly relief pain in patients with CMP.

### Effects of duloxetine in the interference of pain and quality of life

**Fig. 4:**
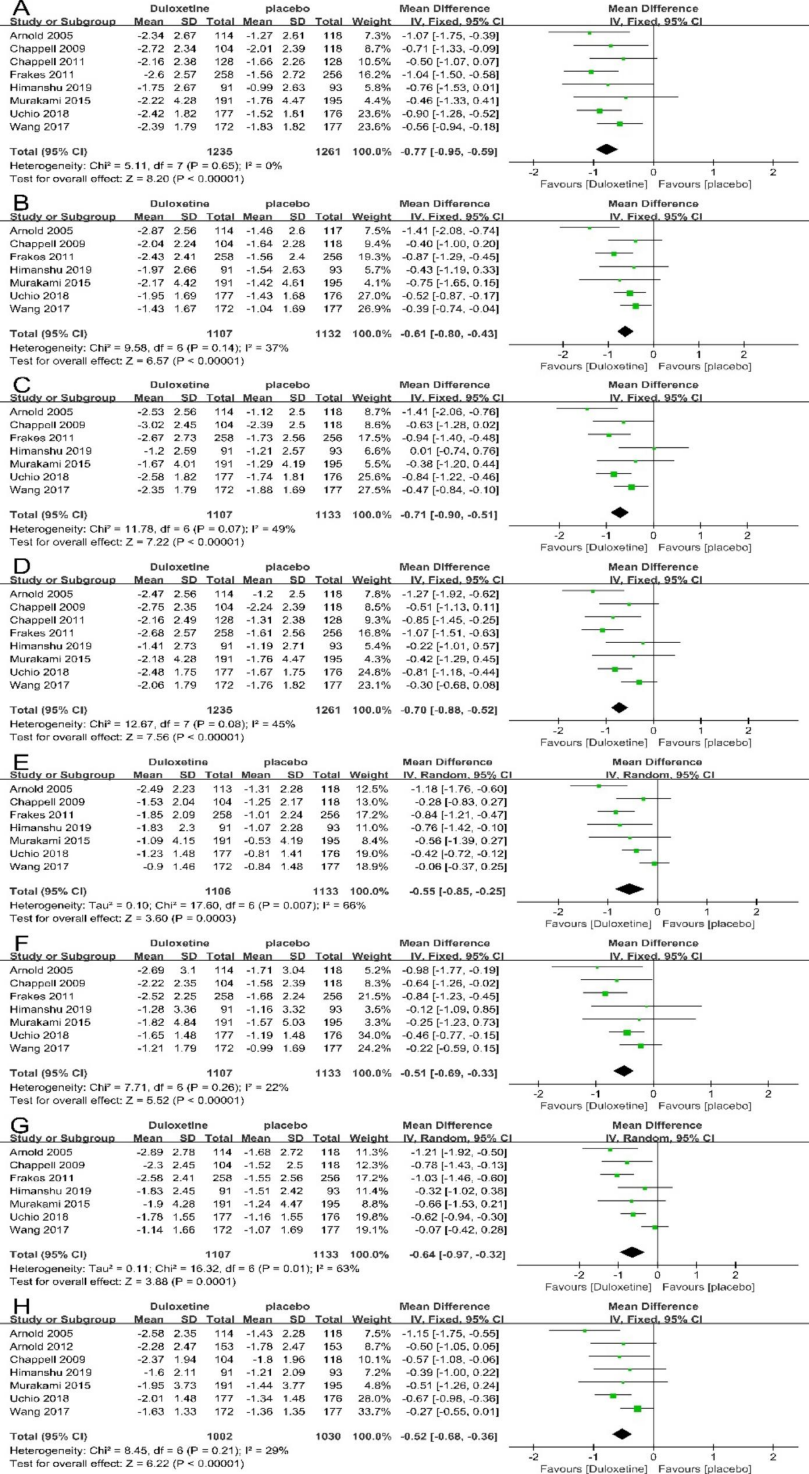
Forest plot: Effects of duloxetine in the interference of pain and quality of life measured by BPI-I: (A)general activity; (B)mood; (C) walking ability; (D) normal work; (E) relations with other people; (F) sleep; (G) enjoyment of life; (H) average interference. The black horizontal lines represent the 95% confidence intervals, while the squares represent the point estimate. The black diamond represents the overall point estimate and 95% confidence intervals

Eight trials contributed to the meta-analysis of the interference of pain and quality of life based on the BPI-I. The meta-analysis results revealed that the duloxetine group showed prominent improvement in the interference of pain on general activity (8 articles; 2496 patients; MD= -0.77; 95% CI, -0.95 to -0.59; P<0.00001) (Fig. 4A), mood (7 articles; 2239 patients; MD= -0.61; 95% CI, -0.80 to -0.43; P<0.00001) (Fig. 4B), walking ability (7 articles; 2240 patients; MD= -0.71; 95% CI, -0.90 to -0.51; P<0.00001) (Fig. 4C), normal work (8 articles; 2496 patients; MD= -0.70; 95% CI, -0.88 to -0.52; P<0.00001) (Fig. 4D), interpersonal relationship (7 articles; 2239 patients; MD= -0.55; 95% CI, -0.85 to -0.25; P = 0.0003) (Fig. 4E), sleep (7 articles; 2240 patients; MD= -0.51; 95% CI, -0.69 to -0.33; P<0.00001) (Fig. 4F), enjoyment of life (7 articles; 2240 patients; MD= -0.64; 95% CI, -0.97 to -0.32; P = 0.0001) (Fig. 4G) and average interference (7 articles; 2032 patients; MD= -0.52; 95% CI, -0.68 to -0.36; P<0.00001) (Fig. 4H) than placebo control group, which indicated that duloxetine can improve the interference of pain, depressive symptoms and the quality of life significantly.

### Effects of duloxetine on the physical function

**Fig. 5:**
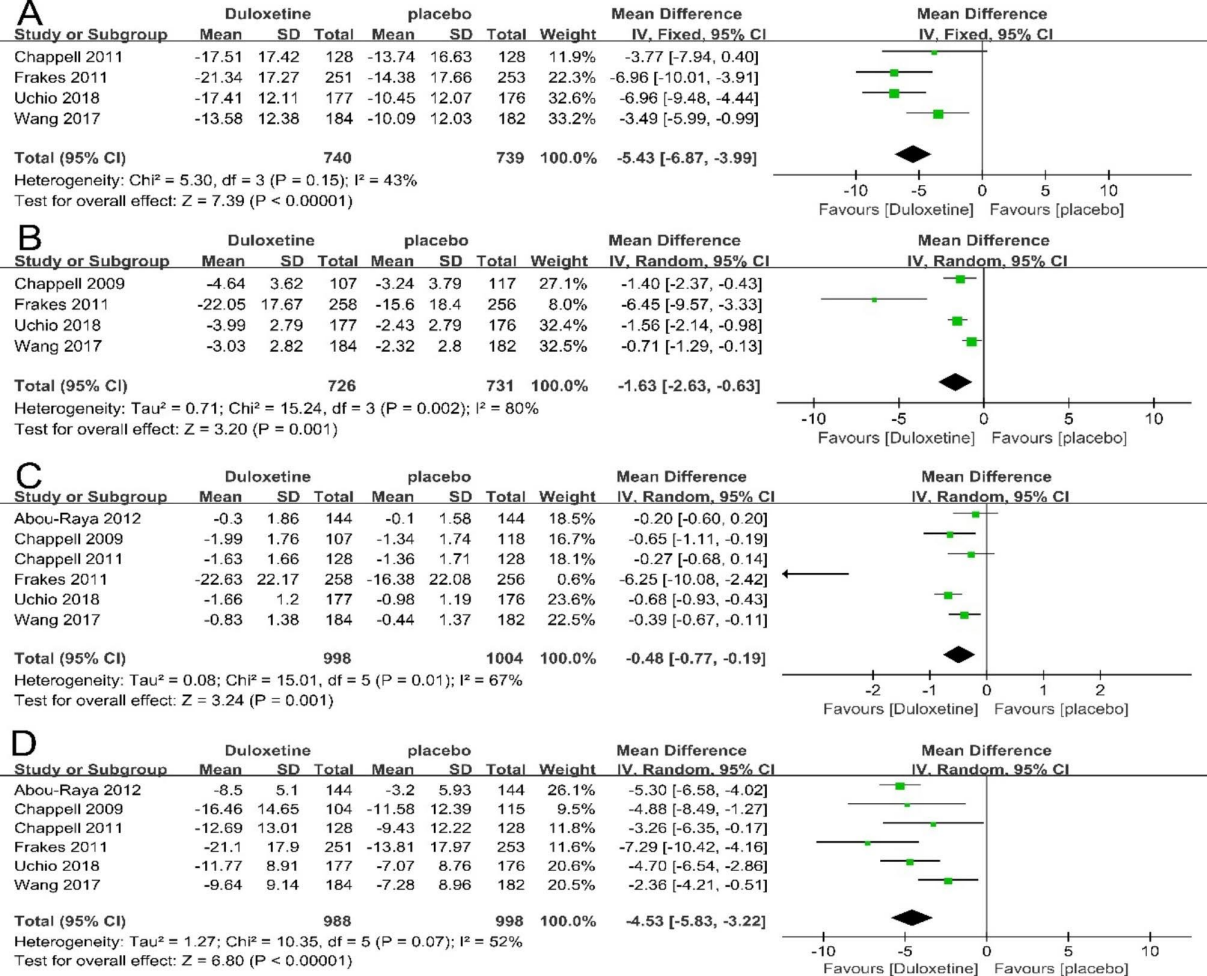
Forest plot: Effects of duloxetine on the physical function measured by WOMAC: (A)total subscale;(B) pain subscale; (C) stiffness subscale;(D) physical function subscale. The black horizontal lines represent the 95% confidence intervals, while the squares represent the point estimate. The black diamond represents the overall point estimate and 95% confidence intervals

Six trials were included in the mate-analysis of WOMAC subscale, and these studies showed that duloxetine can significantly improve the total score of limb function (4 articles; 1479 patients; MD= -5.43; 95% CI, -6.87 to -3.99; P<0.00001) (Fig. 5A), pain (4 articles; 1457 patients; MD= -1.63; 95% CI, -2.63 to -0.63; P = 0.001) (Fig. 5B), stiffness (6 articles; 2002 patients; MD= -0.48; 95% CI, -0.77 to -0.19; P = 0.001) (Fig. 5C), physical function (6 articles; 1996 patients; MD= -4.53; 95% CI, -5.83 to -3.22; P<0.00001) (Fig. 5D) than placebo control. These evidences showed that duloxetine can improve the physical function for the patients with CMP.

### Effects of duloxetine in patient’s global impression

**Fig. 6:**
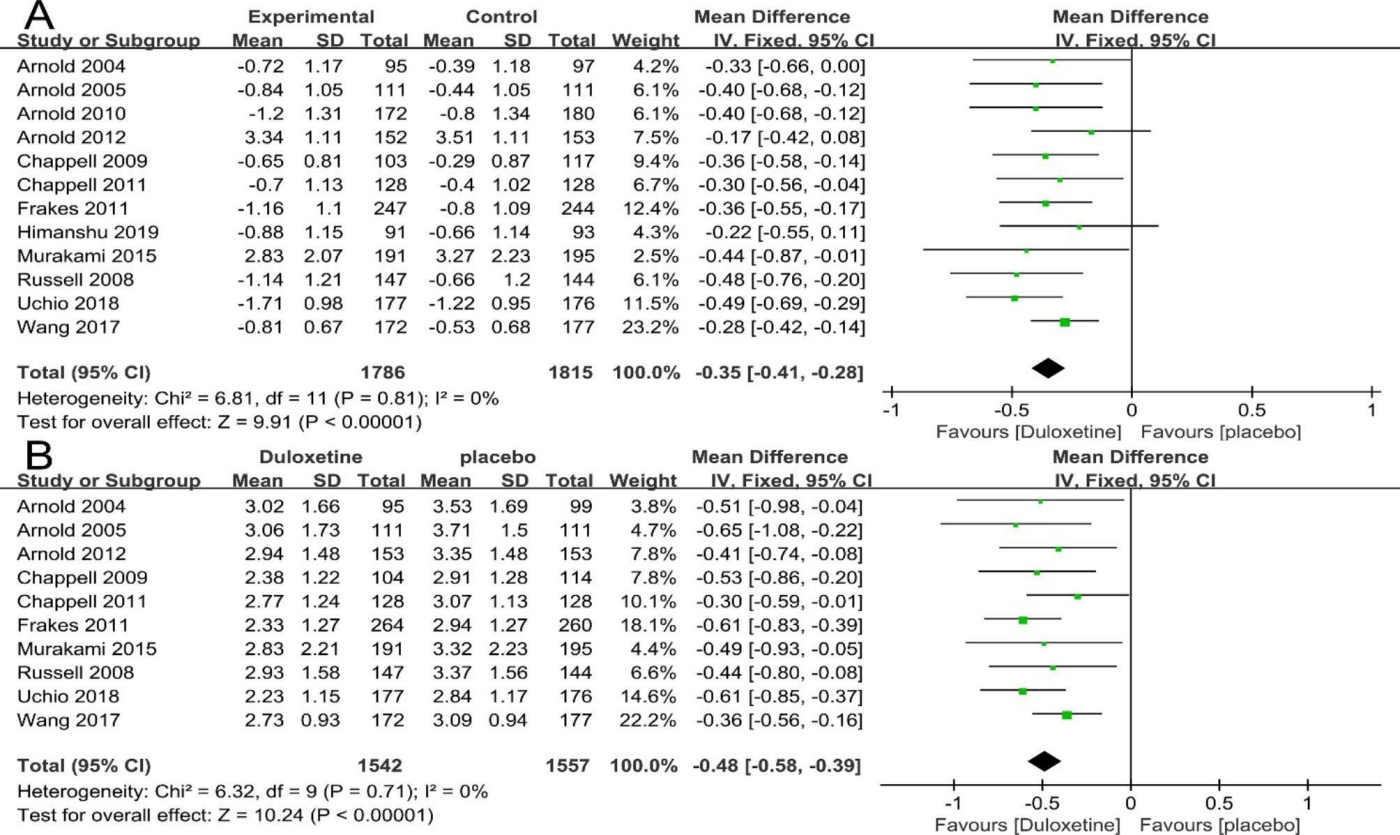
Forest plot: Effects of duloxetine in patient’s global impression : (A)CGI-S; (B)PGI-I. The black horizontal lines represent the 95% confidence intervals, while the squares represent the point estimate. The black diamond represents the overall point estimate and 95% confidence intervals

This meta-analysis revealed that duloxetine had improvement of patient’s global impression significantly than placebo control measured by CGI-S (12 articles; 3601 patients; MD= -0.35; 95% CI, -0.41 to -0.28; P<0.00001) (Fig. 6A) and PGI-I (10 articles; 3099 patients; MD= -0.48; 95% CI, -0.58 to -0.39; P<0.00001) (Fig. 6B).

### Safety

**Fig. 7:**
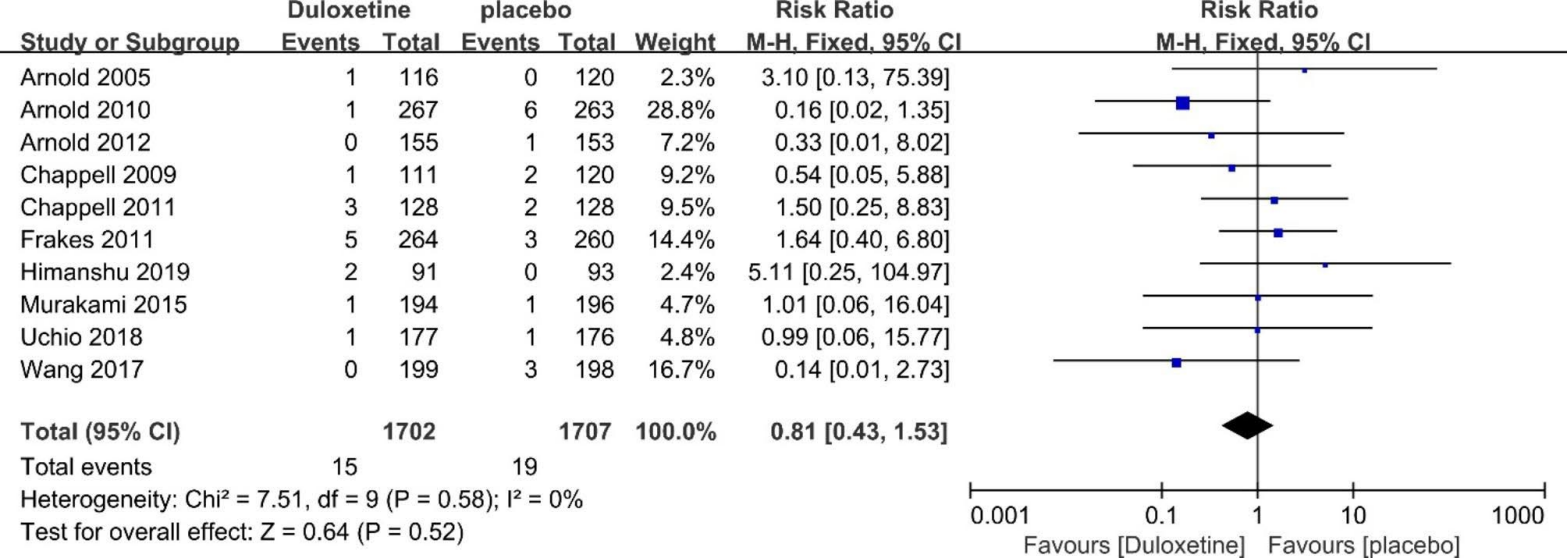
Forest plot: Effects of duloxetine in patient’s safety: SAEs. The black horizontal lines represent the 95% confidence intervals, while the squares represent the point estimate. The black diamond represents the overall point estimate and 95% confidence intervals

Ten trials described the number of serious adverse reactions. The results of this meta-analysis showed that there was no significant difference in the rate of SAEs between duloxetine and placebo groups (10 trials; 3409 patients; RR = 0.81; 95% CI, 0.43 to 1.53; P = 0.52) (Fig. 7).

### Sensitivity analysis

To validate the reliability of results, each of the 13 studies was sequentially eliminated one by one, and sensitivity analysis of the remaining studies was conducted at each step. The sensitivity analysis showed that no individual study significantly influenced the results, indicating the robust result of this meta-analysis. Sensitivity analysis images can be found in the Additional file 3.

### Publication bias

**Fig. 8:**
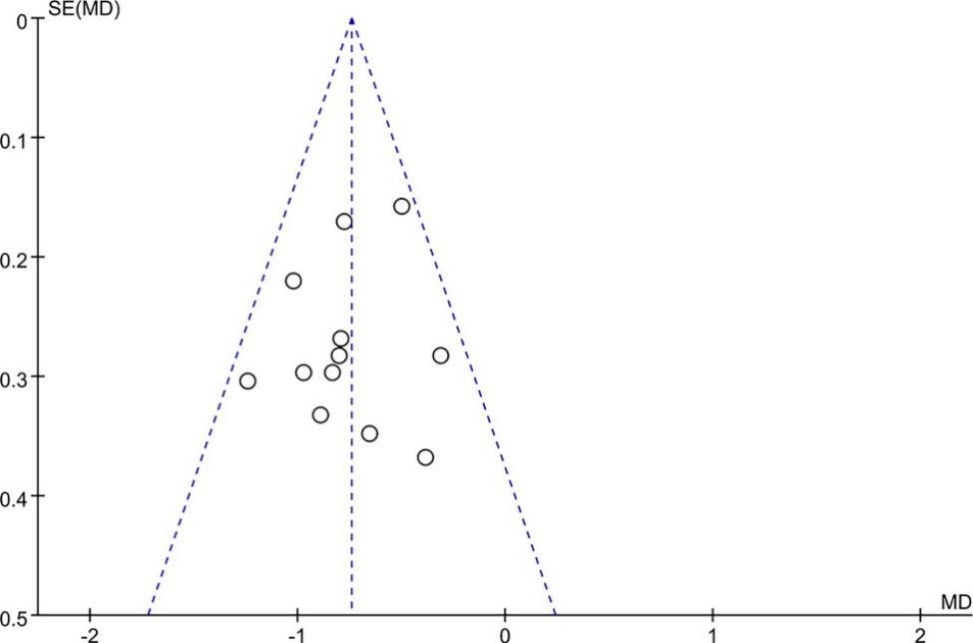
Funnel plot: Publication bias of BPI 24-h average pain. No significant funnel asymmetry that could indicate publication bias was observed

We used funnel plot and Egger’s test to detect publication bias in the outcome of the BPI-S 24 h average pain. No distinct asymmetry could be observed from the shape of funnel plot, suggesting no proof of publication bias (p = 0.492) (Fig. 8).

## Discussion

Consistent with previous studies, the results of this meta-analysis showed that duloxetine can significantly reduce the patient’s pain level. It can also effectively improve patient’s mood and quality of life, which may be related to its pain relief effect, and delay the progress of chronic musculoskeletal diseases such as knee osteoarthritis and fibromyalgia syndrome and ameliorate the limb function of patients. Thus, duloxetine had a definite efficacy in the treatment of patients with CMP, and the incidence of SAEs was no statistically different compared with placebo control.

Today, CMP has become one of the major problems faced by the older people, which affects their normal work and life seriously. Long-term chronic pain is unfavorable to the patients’ psychology and mood, and may even induce depression, anxiety, Alzheimer or other diseases [32, 33]. Therefore, relieving pain levels is the primary goal of CMP treatment. Some researchers [34] think that chronic pain, as a high-pressure condition, is one of the critical factors for determining depression, and their coexistence tends to further aggravate the severity of both disorders and this may be the result of the fact that physical pain sensation management shares the same brain regions, neurotransmitters, and signaling pathways with emotional management which form a histological structural foundation for the coexistence of pain and depression. The neuroplastic changes caused by chronic pain are the possible mechanisms leading to depression, which is a potentially important route for the onset and aggravation of depression. Several animal experiments and clinical studies have demonstrated that opioids can play a role in antidepressant treatment by modulating the neurotransmitter system [35, 36]. However, the serious dependence and addiction caused by opioids cannot be ignored. Therefore, the use of antidepressant drugs to exert analgesic effect has attracted more and more attention in recent years. Consistent with previous studies [37–39], this study found that duloxetine was effective in reducing patients’ pain, and there was a statistical difference between the experimental group and the control group. In addition to duloxetine, a variety of antidepressant treatment [40, 41] have a positive effect on pain.

More and more studies [42–44] reported that norepinephrine and serotonin, as central descending neurotransmitters, can effectively suppress pain, and their reduction is one of the causes of anxiety and depression. The changes of norepinephrine and serotonin in the central system are thought to be the root cause of the comorbidity of pain and depression. Duloxetine, a norepinephrine and serotonin reuptake inhibitor (NSRI), can increase the levels of norepinephrine and serotonin in the central nervous system and play an antidepressant and analgesic role. This study also proved that, on the index of mood improvement, the depression symptoms of patients with CMP treated with duloxetine were significantly reduced, and they were more active in normal interpersonal communication and work life than patients with placebo control group.

The reduction of patients’ pain is conducive to the improvement of patients’ global impression. This study found that the evaluation of patients’ global efficacy and global evaluation showed the same trend as the pain score, indicating that duloxetine has obvious efficacy for CMP and can effectively improve patients’ global impression. There is a significant difference between the experimental group and the control group.

NSAIDs are currently the first-line treatment for the pain with CMP. However, Enthoven [45] demonstrated that only six of 13 RCTs included in the Cochrane review showed that NSAIDs are more effective than placebo in regard to pain improvement. Furthermore, a systematic review by Castellsague [46] also reported a higher risk of gastrointestinal complications associated with NSAIDs as compared to placebo. This systematic review found no differences in SAEs between the duloxetine and placebo groups. Meanwhile, the results are also consistent with the most recent meta-analysis [13, 47, 48]. These results demonstrate that duloxetine has the highest efficacy for reducing pain while minimizing minor adverse effects.

There are several limitations of meta-analysis that should be taken into account. First, the meta-analysis only included English literature, and based on a relatively small number of RCTs, which may lead to bias risk. Fortunately, the quality of trials we included was relatively high. Second, the included RCTs lack long-term follow-up studies, so the long-term efficacy and safety of duloxetine are still unclear. The included RCTs mostly used placebo as a control and lacked comparison with NSAIDs. Also, the current systematic review was not registered, which is a deficiency of this study. Lastly, in this study and most of the current meta-analyses, the statistical model of Meta-analysis (fixed or random effect method) is based on the results of statistical test of heterogeneity, which has certain shortcomings.

## Conclusion

In conclusion, antidepressant drugs like duloxetine, is an effective treatment option for patients with CMP. Antidepressant treatment is a non-negligible factor in the treatment of CMP. This study improved that duloxetine can significantly reduce the pain level of patients, improve depressive symptoms and global impression, and has no obvious serious adverse reactions.

Any drug has two sides, and we should explore the potential issues of duloxetine in the treatment of CMP in the future. Further studies should confirm the relationship between psychological diseases and CMP, explore its internal relationship, and verify the efficacy and safety of antidepressant drugs for musculoskeletal Diseases.

## Electronic supplementary material

Below is the link to the electronic supplementary material.

Supplementary Material 1

Supplementary Material 2

Supplementary Material 3

## Data Availability

All data generated or analysed during this study are included in this published article.

## Abbreviations

CMP: Chronic musculoskeletal pain
RCTs: Randomized controlled trials
SNRI: serotonin and norepinephrine reuptake inhibitor
MD: mean difference
CI: confident interval
RR: relative risk
BPI-S: Brief Pain Inventory-Severity
BPI-I: Brief Pain Inventory-Interference
WOMAC: Western Ontario and McMaster Universities Osteoarthritis Index
SF-36: 36-Item Short-Form Health Status Survey
CGI-S: Clinical Global Impressions of Severity
PGI-I: Patient’s Global Impression of Improvement
SAEs: serious adverse events
KOA: Knee osteoarthritis
NSAIDs: non-steroidal anti-inflammatory drugs
