# Supplementary material for: Efficacy and safety of duloxetine in chronic musculoskeletal pain: a systematic review and meta-analysis
Source: BMC Musculoskelet Disord. 2023 May 18;24:394. doi: 10.1186/s12891-023-06488-6 (PMC10193733; doi:10.1186/s12891-023-06488-6)

**Additional file 1.** Search strategies for Pubmed databases searched.

**#1** ((duloxetine [Title/Abstract]) OR duloxetine hydrochloride [Title/Abstract])

**#2** ((((((chronic musculoskeletal pain [Title/Abstract]) OR CMP [Title/Abstract]) OR osteoarthritis [Title/Abstract]) OR OA [Title/Abstract]) OR fibromyalgia [Title/Abstract]) OR FM [Title/Abstract])

**#3** (#1) AND (#2)

**#4** limit #3 to humans

**#5** limit #4 to time from 1000/01/01-2022/05/31

**Search details:** (((duloxetine [Title/Abstract]) OR duloxetine hydrochloride [Title/Abstract]) AND ((((((chronic musculoskeletal pain [Title/Abstract]) OR CMP [Title/Abstract]) OR osteoarthritis [Title/Abstract]) OR OA [Title/Abstract]) OR fibromyalgia [Title/Abstract]) OR FM [Title/Abstract]) AND (human [All Fields]))

**Filters:** from 1000/1/1 - 2022/5/31


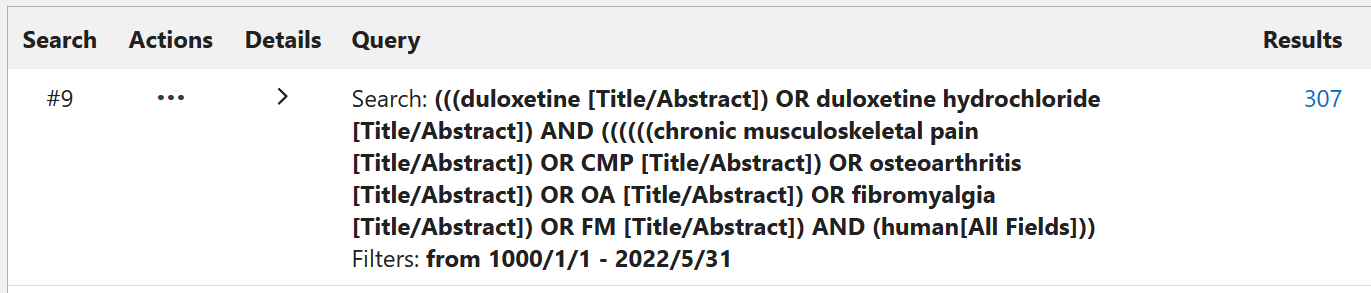

Supplement: Supplementary file 1 — Supplementary Material 1 [file 12891_2023_6488_MOESM1_ESM.docx]
