# Supplementary material for: Efficacy and safety of duloxetine in chronic musculoskeletal pain: a systematic review and meta-analysis
Source: BMC Musculoskelet Disord. 2023 May 18;24:394. doi: 10.1186/s12891-023-06488-6 (PMC10193733; doi:10.1186/s12891-023-06488-6)
Supplement: Supplementary file 2 — Supplementary Material 2 [file 12891_2023_6488_MOESM2_ESM.docx]

**Additional file 2.** Studies excluded, including reasons for exclusion

| Article | Reference | Reasons for exclusion |
| --- | --- | --- |
| Garcia-Campayo, 2009 | Garcia-Campayo J., Serrano-Blanco A., Rodero B., Magallon R., Alda M., Andres E., Luciano J. V., del Hoyo Y. L. Effectiveness of the psychological and pharmacological treatment of catastrophization in patients with fibromyalgia: a randomized controlled trial. Trials, 2009. 10:24.https://doi.org/ 10.1186/1745-6215-10-24. | Inappropriate Intervention |
| Chappell, 2008 | Chappell A. S., Bradley L. A., Wiltse C., Detke M. J., D'Souza D. N., Spaeth M. A six-month double-blind, placebo-controlled, randomized clinical trial of duloxetine for the treatment of fibromyalgia. Int J Gen Med, 2008. 1:91-102.https://doi.org/ 10.2147/ijgm.s3979. | Inappropriate Outcomes |
| Sullivan Mark, 2009 | Sullivan Mark D., Bentley Susan, Fan Ming-Yu, Gardner Greg. A single-blind, placebo run-in study of duloxetine for activity-limiting osteoarthritis pain. The journal of pain, 2009. 10(2):208-213.https://doi.org/ 10.1016/j.jpain.2008.08.009. | Inappropriate Outcomes |
| Henry, 2018 | Henry N. Lynn, Unger Joseph M., Schott Anne F., Fehrenbacher Louis, Flynn Patrick J., Prow Debra M., Sharer Carl W., Burton Gary V., Kuzma Charles S., Moseley Anna, Lew Danika L., Fisch Michael J., Moinpour Carol M., Hershman Dawn L., Wade James L., 3rd. Randomized, Multicenter, Placebo-Controlled Clinical Trial of Duloxetine Versus Placebo for Aromatase Inhibitor-Associated Arthralgias in Early-Stage Breast Cancer: SWOG S1202. Journal of clinical oncology : official journal of the American Society of Clinical Oncology, 2018. 36(4):326-332.https://doi.org/ 10.1200/JCO.2017.74.6651. | Inappropriate Outcomes |
| van den Dries, 2022 | van den Driest J. J., Schiphof D., Koffeman A. R., Koopmanschap M. A., Bindels P. J. E., Bierma-Zeinstra S. M. A. No Added Value of Duloxetine in Patients With Chronic Pain due to Hip or Knee Osteoarthritis: A Cluster-Randomized Trial. Arthritis Rheumatol, 2022. 74(5):818-828.https://doi.org/ 10.1002/art.42040. | Inappropriate Comparison Group |
| Wang, 2019 | Wang G., Bi L., Li X., Li Z., Zhao D., Chen J., He D., Wang C. N., Wu T., Duenas H., Skljarevski V., Yue L. Maintenance of effect of duloxetine in Chinese patients with pain due to osteoarthritis: 13-week open-label extension data. BMC Musculoskelet Disord, 2019. 20(1):174.https://doi.org/ 10.1186/s12891-019-2527-y. | Inappropriate Intervention |
| Williamson, 2014 | Williamson O. D., Schroer M., Ruff D. D., Ahl J., Margherita A., Sagman D., Wohlreich M. M. Onset of response with duloxetine treatment in patients with osteoarthritis knee pain and chronic low back pain: a post hoc analysis of placebo-controlled trials. Clin Ther, 2014. 36(4):544-551.https://doi.org/ 10.1016/j.clinthera.2014.02.009. | Inappropriate study design |
| Bidari, 2019 | Bidari A., Moazen-Zadeh E., Ghavidel-Parsa B., Rahmani S., Hosseini S., Hassankhani A. Comparing duloxetine and pregabalin for treatment of pain and depression in women with fibromyalgia: an open-label randomized clinical trial. Daru, 2019. 27(1):149-158.https://doi.org/ 10.1007/s40199-019-00257-4. | Inappropriate Intervention |
| Gilron, 2016 | Gilron I., Chaparro L. E., Tu D., Holden R. R., Milev R., Towheed T., DuMerton-Shore D., Walker S. Combination of pregabalin with duloxetine for fibromyalgia: a randomized controlled trial. Pain, 2016. 157(7):1532-1540.https://doi.org/ 10.1097/j.pain.0000000000000558. | Inappropriate Intervention |
| Enomoto, 2018 | Enomoto H., Fujikoshi S., Tsuji T., Sasaki N., Tokuoka H., Uchio Y. Efficacy of duloxetine by prior NSAID use in the treatment of chronic osteoarthritis knee pain: A post hoc subgroup analysis of a randomized, placebo-controlled, phase 3 study in Japan. J Orthop Sci, 2018. 23(6):1019-1026.<https://doi.org/> 10.1016/j.jos.2018.07.008. | Inappropriate study design |
| Gaynor, 2011 | Gaynor P. J., Gopal M., Zheng W., Martinez J. M., Robinson M. J., Marangell L. B. A randomized placebo-controlled trial of duloxetine in patients with major depressive disorder and associated painful physical symptoms. Curr Med Res Opin, 2011. 27(10):1849-1858.https://doi.org/ 10.1185/03007995.2011.609539. | Inappropriate Outcomes |
