# Supplementary material for: Efficacy and safety of duloxetine in chronic musculoskeletal pain: a systematic review and meta-analysis
Source: BMC Musculoskelet Disord. 2023 May 18;24:394. doi: 10.1186/s12891-023-06488-6 (PMC10193733; doi:10.1186/s12891-023-06488-6)
Supplement: Supplementary file 3 — Supplementary Material 3 [file 12891_2023_6488_MOESM3_ESM.docx]

**Additional file 3.** Sensitivity analysis results


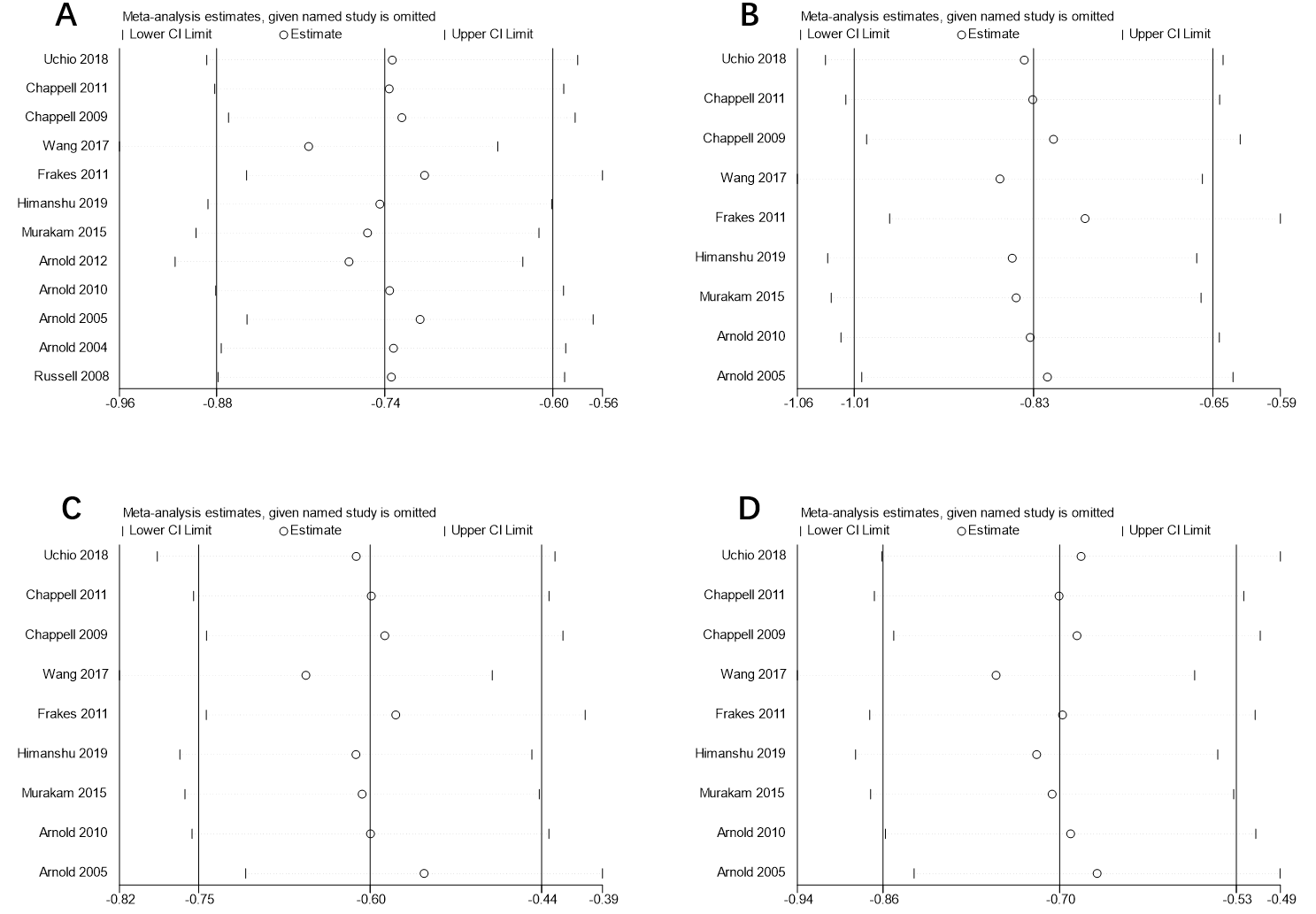


**Figure S1** Sensitivity analysis of BPI−S: (A) average pain; (B) worst pain; (C) least pain; (D) pain right now.


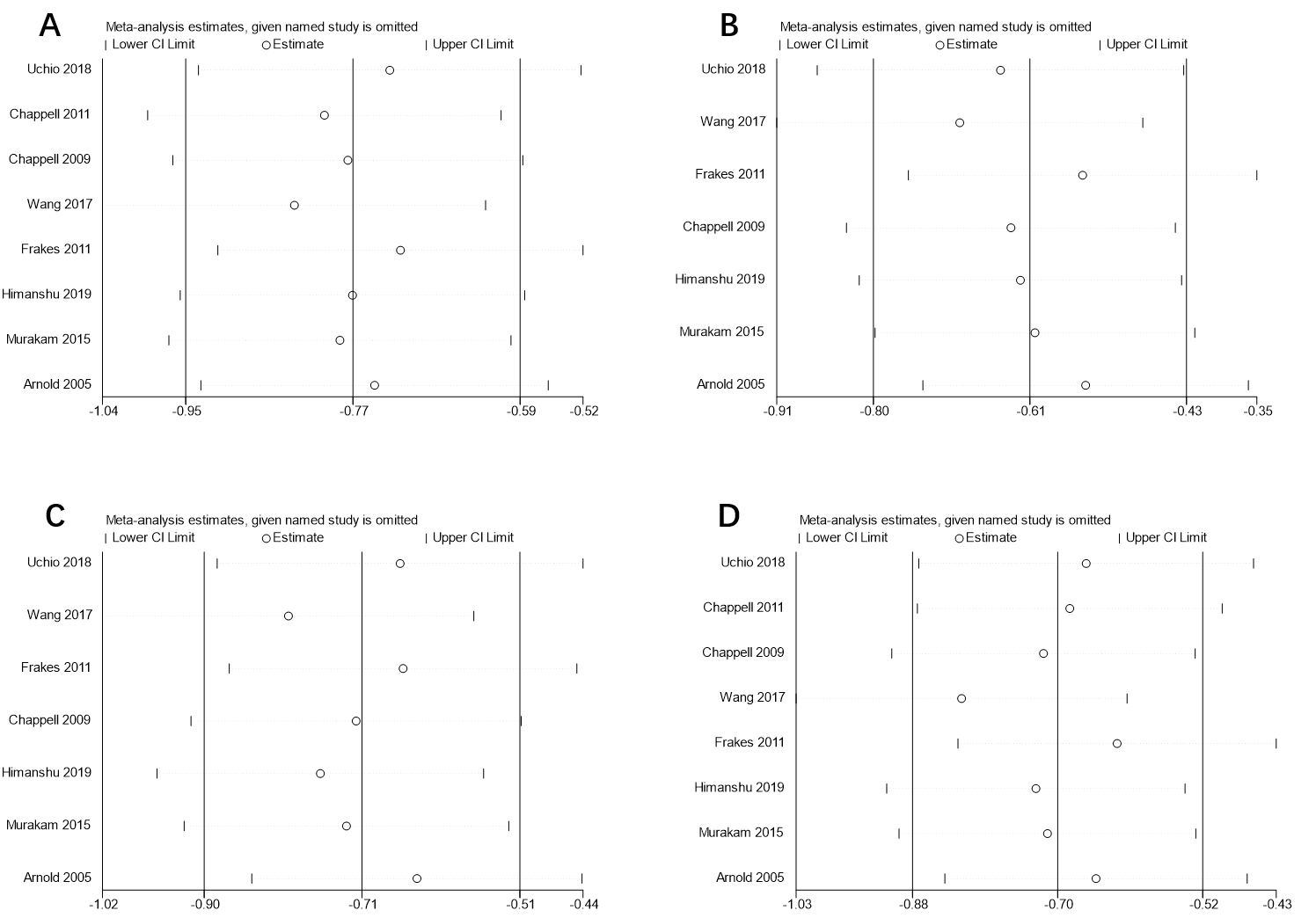

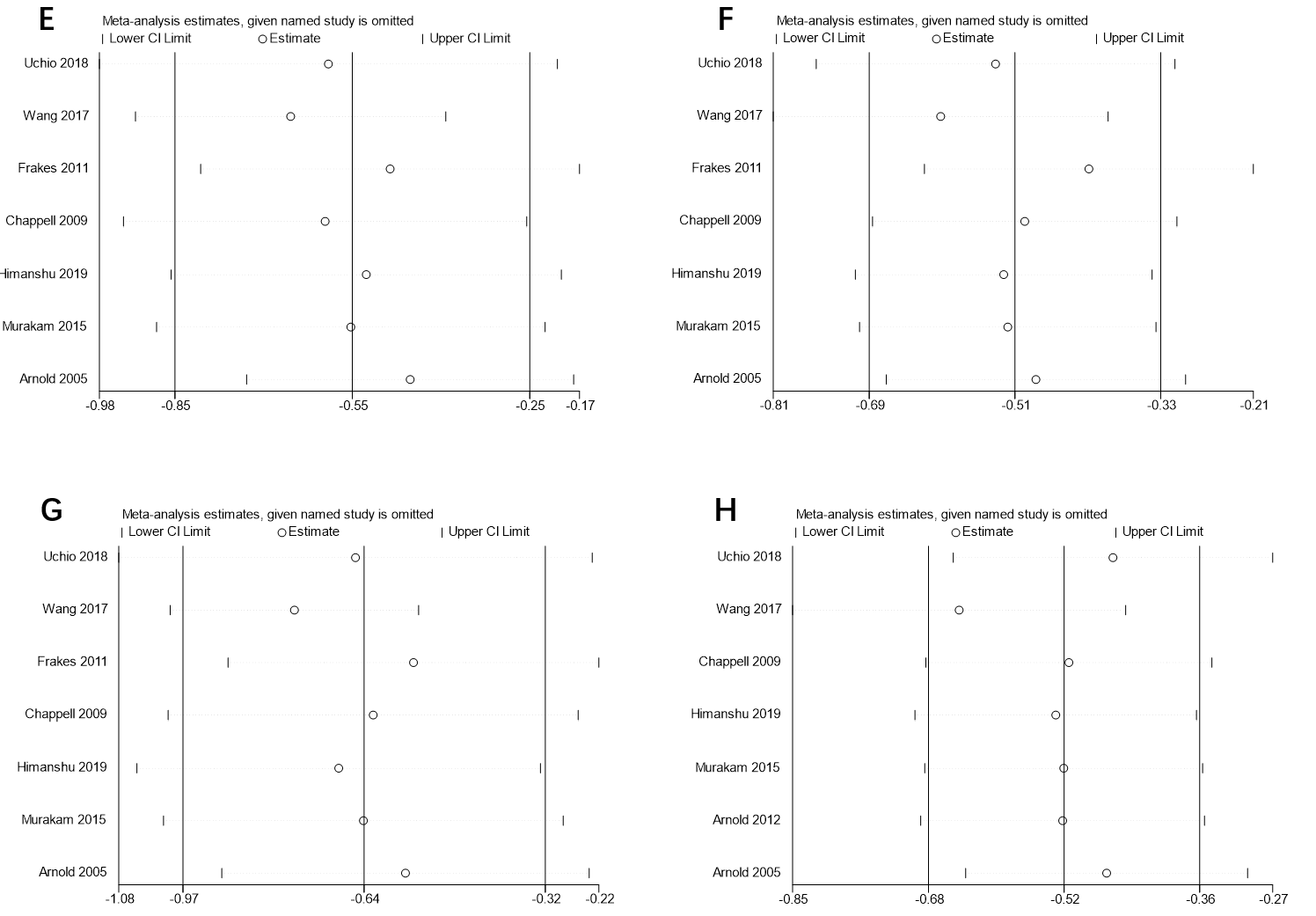
**Figure S2** Sensitivity analysis of BPI−I: (A) general activity; (B) mood; (C) walking ability;(D) normal work; (E) relations with other people; (F) sleep; (G) enjoyment of life; (H) average interference.


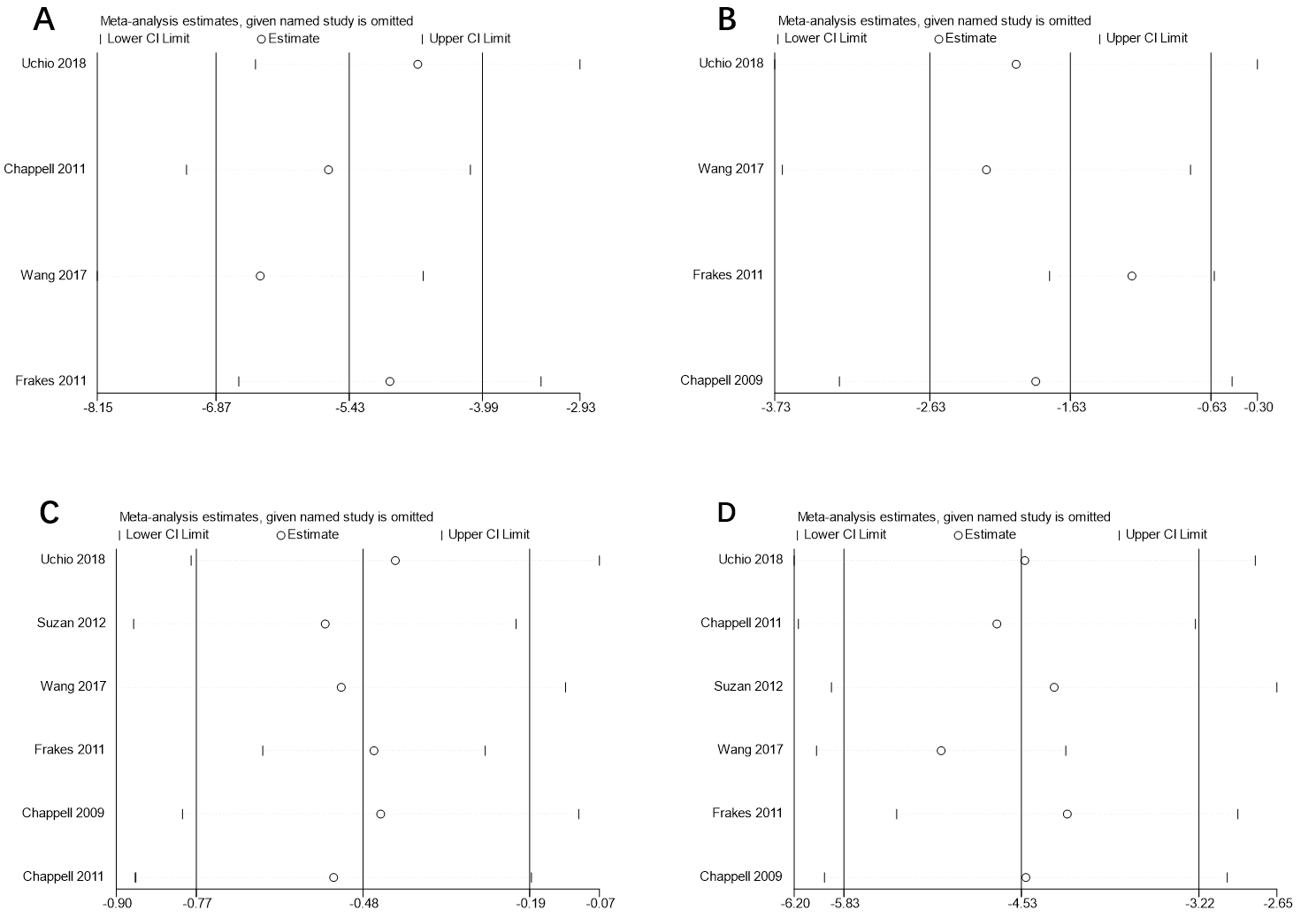


**Figure S3** Sensitivity analysis of WOMAC: (A) total subscale; (B) pain subscale; (C) stiffness subscale; (D) physical function subscale.


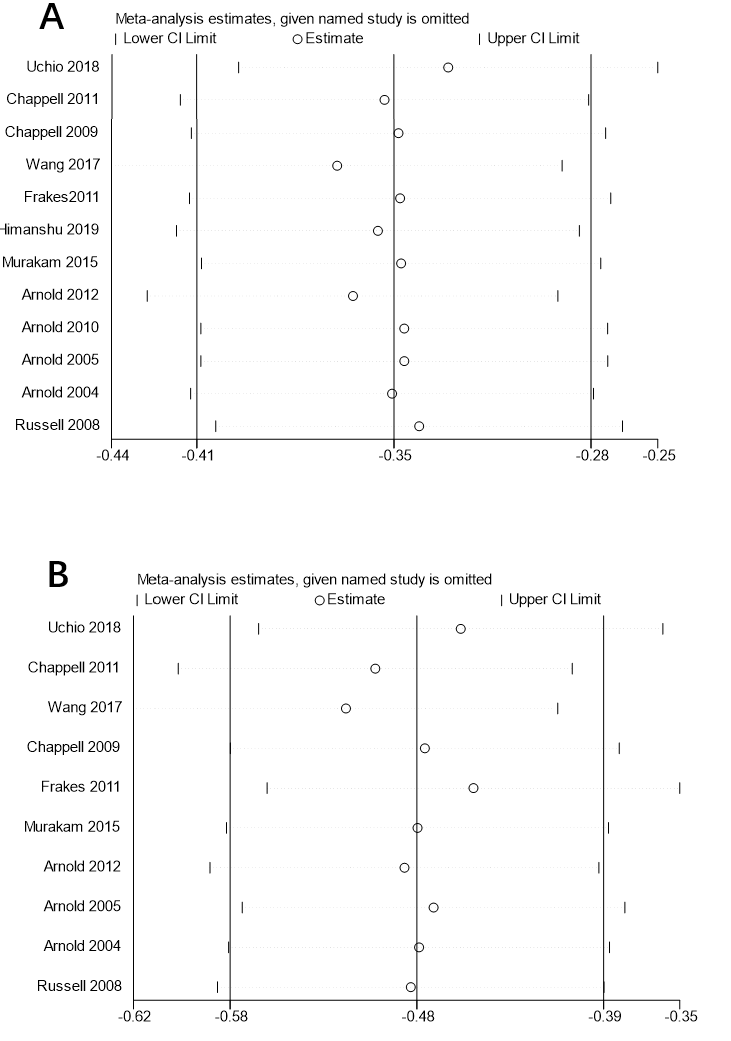


**Figure S4** Sensitivity analysis: (A) Sensitivity analysis of CGI-S; (B) Sensitivity analysis of PGI-I.

**Figure S5** Sensitivity analysis of SAEs.
